# Anti-Photoaging Effects of Upcycled *Citrus junos* Seed Anionic Peptides on Ultraviolet-Radiation-Induced Skin Aging in a Reconstructed Skin Model

**DOI:** 10.3390/ijms25031711

**Published:** 2024-01-30

**Authors:** Hyun-Ju Ko, Su-An Sim, Mi-Hee Park, Hwa-Sun Ryu, Won-Yeong Choi, Sung-Min Park, Jung-No Lee, Chang-Gu Hyun

**Affiliations:** 1 Bio Convergence R&D Center, CoSeedBioPharm Corporation, Heungdeok-gu, Cheongju 28161, Republic of Korea; ok6336@hanmail.net (H.-J.K.); mhgoogt@hanmail.net (M.-H.P.); hwasun157@hanmail.net (H.-S.R.); cww6341@naver.com (W.-Y.C.); smp@coseed.co.kr (S.-M.P.); 2 Department of Beauty and Cosmetology, Jeju Inside Agency and Cosmetic Science Center, Jeju National University, Jeju 63243, Republic of Korea

**Keywords:** upcycling, byproducts, citron seed anionic peptide, reconstructed skin model

## Abstract

Side streams and byproducts of food are established sources of natural ingredients in cosmetics. In the present study, we obtained upcycled low-molecular-weight anionic peptides (LMAPs) using byproducts of the post-yuzu-juicing process by employing an enzyme derived from *Bacillus* sp. For the first time, we isolated anionic peptides less than 500 Da in molecular weight from *Citrus junos* TANAKA seeds via hydrolysis using this enzyme. The protective effect of LMAPs against UVR-induced photoaging was evaluated using a reconstructed skin tissue (RST) model and keratinocytes. The LMAPs protected the keratinocytes by scavenging intracellular reactive oxygen species and by reducing the levels of paracrine cytokines (IL-6 and TNF-α) in UVR (UVA 2 J/cm^2^ and UVB 15 mJ/cm^2^)-irradiated keratinocytes. Additionally, the increase in melanin synthesis and TRP-2 expression in RST caused by UVR was significantly inhibited by LMAP treatment. This treatment strongly induced the expression of filaggrin and laminin-5 in UVR-irradiated RST. It also increased type I collagen expression in the dermal region and in fibroblasts in vitro. These results suggest that a hydrolytic system using the enzyme derived from *Bacillus* sp. can be used for the commercial production of LMAPs from food byproducts and that these LMAPs can be effective ingredients for improving photoaging-induced skin diseases.

## 1. Introduction

The growing importance of sustainable environmental, social, and governance (ESG) management has encouraged the upcycling of food byproducts as raw materials in the cosmetics industry. Food side streams and byproducts are established sources of natural ingredients in cosmetics. Rising consumer demand has thus increased the need for such raw materials in the manufacturing of eco-friendly and natural personal-care products.

*Citrus junos* TANAKA (yuzu; citron), an evergreen broad-leaved shrub belonging to the family Rutaceae, is a citrus plant found in East Asia [1,2]. Yuzu seeds account for 10–20% of their pulp weight and are a rich source of limonoids and bioflavonoids. In addition, they contain several compounds with antioxidant properties, indicating their potential as functional materials [3,4,5]. Yuzu seeds are an excellent source of lipids and are primarily used for the production of oils and fats. Furthermore, the byproducts from the oil manufacturing process, discarded because of their bitter taste, could serve as raw materials for the isolation of active substances. However, although citron seeds contain approximately 10–20% and 20–30% (by weight) of crude protein and oil/fat components, respectively, there are only limited indications for their use [6].

Biological peptides can be extracted from various natural resources such as plants, animals, and microorganisms. These peptides have been proven to have various physiological effects, such as antioxidant, anti-aging, moisturizing, collagen-stimulating, and wound-healing effects, in in vitro and ex vivo experiments and clinical trials [7]. Among peptides with these functions, the most biologically powerful ones generally consist of 2–9 amino acids and have a molecular weight of <3000 Da [8].

The human skin possesses various molecular mechanisms that protect it from extrinsic factors, including ultraviolet radiation (UVR). The first is the epidermal layer, also called the skin barrier, which acts as the first line of defense against harmful external agents. Another line of protection for the skin is melanocytes. Melanin, a pigment synthesized in these cells, absorbs UVR and prevents it from penetrating into the lower layers of the epidermis [9]. Some stress factors have been shown to affect different cell signaling and biochemical pathways in the skin; for example, UV not only triggers mechanisms that protect the integrity of the skin and regulate the overall internal environmental balance but also triggers skin pathology (aging, cancer, autoimmune reactions) [10]. Long-term exposure to UVR, especially direct exposure to UVA and UVB, is known to damage the structural integrity and physiological function of the skin. UVA radiation negatively affects epidermal keratinocytes and dermal fibroblast and induces long-term changes. UVB radiation-induced changes are visible mainly within the epidermis, but it also penetrates the upper part of dermis. These changes are collectively referred to as skin photoaging [9,11]. The clinical signs of photoaging induced by UVR include wrinkles, rough skin, skin barrier damage, and hyperpigmentation in the skin [12]. These signs appear due to increased oxidative stress, upregulation of the expression of matrix metalloproteinases (MMPs), breakdown of the extracellular matrix (ECM), and inflammatory response in UVR-exposed skin [13]. Oxidative stress is particularly known to be involved in the progression of photoaging and is considered a key trigger in the early stages of photoaging. Reactive oxygen species (ROSs) activate the expression of inflammation-related cytokines, including interleukin (IL)-1, IL-6, and tumor necrosis factor (TNF)-α, in keratinocytes [14]. UV irradiation results in fibroblast senescence, as well as in decreased collagen production and increased levels of MMPs in fibroblasts, both of which are triggered by proinflammatory cytokines and chemokines released from UV-irradiated keratinocytes [15].

The prevention and regulation of skin photoaging are one of the hotspots in the field of skin and cosmetics research. Currently, compounds isolated from natural resources have been reported to exert protective effects against skin photoaging. Particularly, in recent years, bioactive peptides from various natural resources have gained increasing attention from researchers [16,17,18].

In the present study, we extracted a significant amount of protein from discarded yuzu seeds, which, after decomposition, yielded peptides with amino acid polymers smaller than proteins. Owing to their exceptional skin penetration, we propose the possible use of these low-molecular-weight anionic peptides (LMAPs) in the development of functional materials for manufacturing novel products with excellent physiological activity, safety, and stability. As the use of animals for screening anti-photoaging agents in the medical cosmetics industry is prohibited, we used a reconstructed skin model that mimics human cutaneous tissue. The reconstructed skin model was suitable for studying specific UVR-induced alterations in the epidermis and dermis simultaneously. We evaluated the potential protective effects of citron seed anionic peptides on UVR-induced photoaging using the experimental skin reconstruction model and cells such as keratinocytes, melanoma, and fibroblasts.

## 2. Results

### 2.1. Separation of LMAPs

**Figure 1:**
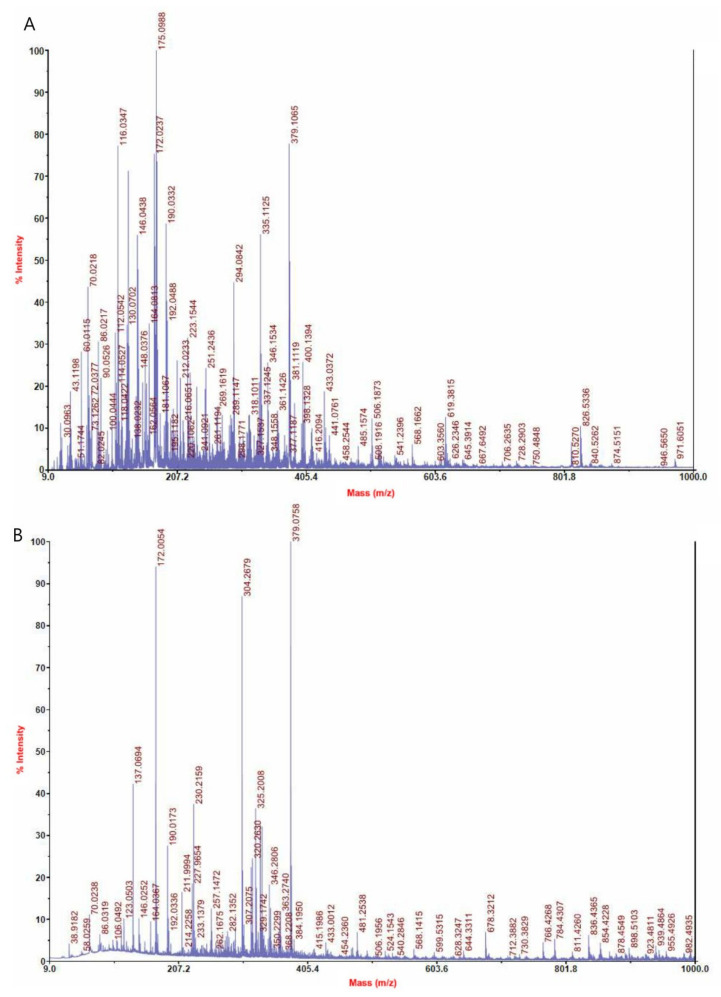
MS spectra of peptides separated from *Citrus junos* TANAKA seeds. (**A**) Low-molecular-weight peptides. (**B**) Low-molecular-weight anionic peptides.

We determined the molecular weight and zeta potential of LMAPs isolated from *C. junos* TANAKA seeds. The molecular weight of the majority of separated peptides was less than 500 Da, suggesting the hydrolysis of the separated peptides into smaller molecules (Figure 1). The surface potential of the peptides separated from the *C. junos* TANAKA seeds is presented in Table 1. The zeta potential of the low-molecular-weight peptides was −0.84 mV and that of the LMAPs was 8.72 mV, indicating that the separation of LMAPs proceeded well using cation exchange resin.

### 2.2. Antioxidant Effects of LMAPs in Keratinocytes

**Figure 2:**
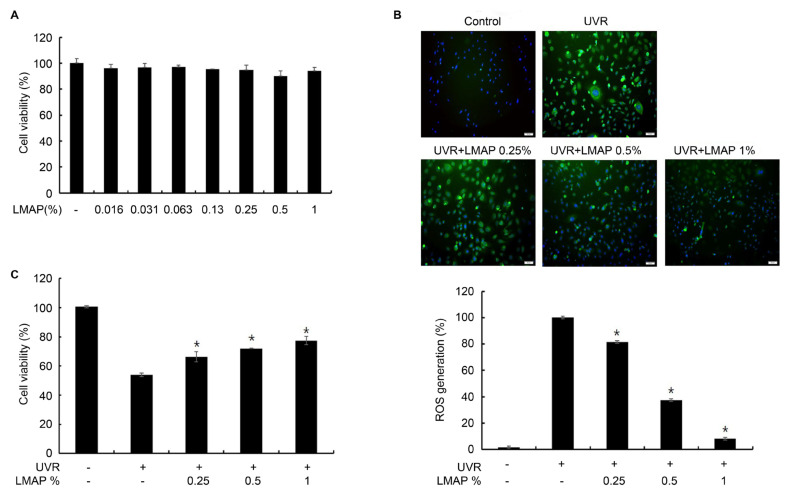
Cytotoxicity of LMAPs in keratinocytes and effects of LMAPs on oxidative stress in UVR-irradiated keratinocytes. (**A**) LMAP treatment was not cytotoxic for keratinocytes. LMAP treatment decreased the levels of UVR-induced intracellular reactive oxygen species (**B**) and cytotoxicity (**C**). Data are presented as mean ± S.D.; *n* = 3; * *p* < 0.05 vs. UVR-irradiated control. LMAP: low-molecular-weight anionic peptide; UVR: UVA 2 J/cm^2^ + UVB 15 mJ/cm^2^. Scale bar, 50 μm.

The LMAPs did not induce significant cell death in the keratinocytes (Figure 2A) to a concentration of 1% (*p* < 0.05; Figure 2A). Therefore, for further experiments, we used 0–1% LMAPs. The intracellular ROS concentration was significantly increased in the UVR-irradiated keratinocytes compared with that in the negative control (*p* < 0.05; Figure 2B). However, LMAP treatment of the UVR-irradiated keratinocytes decreased the intracellular ROS concentration (*p* < 0.05; Figure 2B) and the UVR-induced cytotoxicity (*p* < 0.05; Figure 2C).

### 2.3. LMAP Treatment Inhibits UVR-Induced Cytokine Production by Keratinocytes

**Figure 3:**
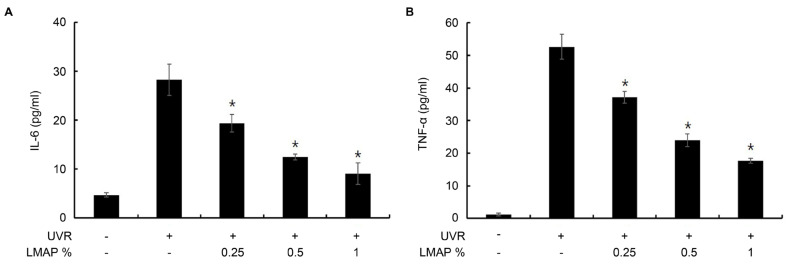
Effect of LMAPs on the levels of cytokines in UVR-irradiated keratinocytes. LMAP treatment decreased UVR-induced secretion of IL-6 (**A**) and TNF-α (**B**) by keratinocytes. Data are presented as mean ± S.D.; *n* = 3; * *p* < 0.05 vs. UVR-irradiated control. LMAP: low-molecular-weight anionic peptide; UVR: UVA 2 J/cm^2^ + UVB 15 mJ/cm^2^.

The levels of TNF-α and IL-6 in the keratinocyte culture medium were significantly increased in the UVR-irradiated keratinocytes. However, their levels were significantly restored in the UVR-irradiated keratinocytes treated with LMAPs (*p* < 0.05; Figure 3).

### 2.4. LMAP Treatment Inhibits Pigmentation following UVR Irradiation

**Figure 4:**
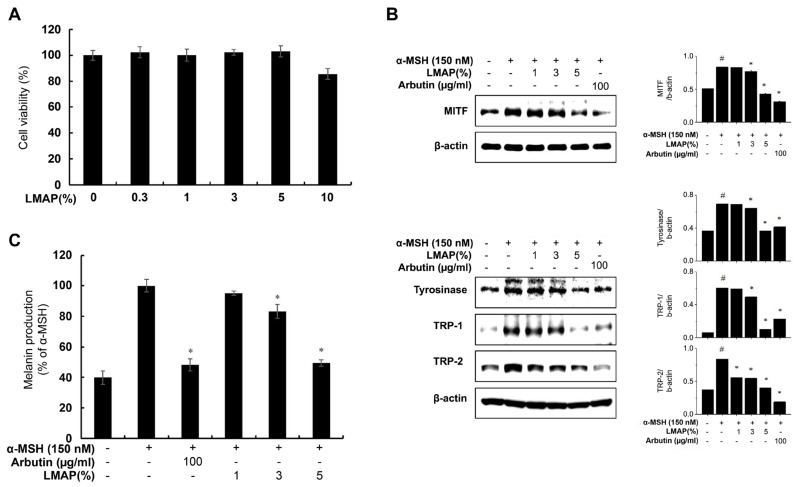
Effect of LMAPs on melanin production and melanogenic protein expression in melanoma cells. LMAP treatment had no effect on the viability of melanoma cells (**A**). LMAPs decreased melanogenic proteins (MITF, TYR, TRP-1, and TRP-2) (**B**) as well as α-MSH-induced melanin pigmentation (**C**). Data are presented as mean ± S.D.; *n* = 3; ^#^ *p* < 0.05 vs. untreated control, * *p* < 0.05 vs. α-MSH-induced control. LMAP: low-molecular-weight anionic peptide.

LMAP treatment had no effect on the viability of melanoma cells (Figure 4A). Treatment with α-melanocyte-stimulating hormone (α-MSH; 150 nM) increased the melanin content of melanoma cells as well as the expression of melanogenic proteins, namely MITF, TYR, and TRP-1,2 compared with that in the negative control (*p* < 0.05; Figure 4B,C). LMAP treatment significantly decreased the melanin content and expression of melanogenic proteins in these cells. LMAPs reduced the melanin content to 83.21% at a 5% concentration, a decrease to a level similar to that of arbutin.

**Figure 5:**
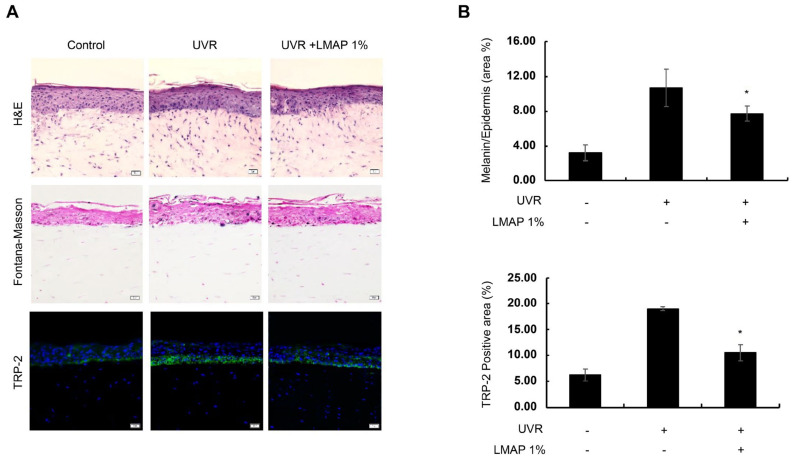
Effect of LMAPs on melanogenesis in the reconstructed skin model following UVR treatment. (**A**) Staining images and (**B**) Calculating graph. LMAPs decreased UVR-induced melanin pigmentation and TRP-2 expression. Data are presented as mean ± S.D.; *n* = 3; * *p* < 0.05 vs. UVR-irradiated control. LMAP: low-molecular-weight anionic peptide; UVR: UVA 2 J/cm^2^ + UVB 15 mJ/cm^2^. Scale bar, 50 μm.

Melanin content was significantly increased in the UVR-irradiated reconstructed skin tissue (RST) compared with that in the negative control (*p* < 0.05; Figure 5). However, LMAP treatment decreased the melanin content following UVR irradiation (*p* < 0.05; Figure 5). Additionally, the expression of tyrosinase-related protein-2 (TRP-2) was significantly decreased in the LMAPs-treated RST compared with that in the untreated UVR-irradiated tissue (*p* < 0.05; Figure 5). Taken together, these results indicate that, following UVR irradiation, LMAP treatment decreases pigmentation in the RST.

### 2.5. LMAP Treatment Inhibits Wrinkle Formation following UVR Irradiation

**Figure 6:**
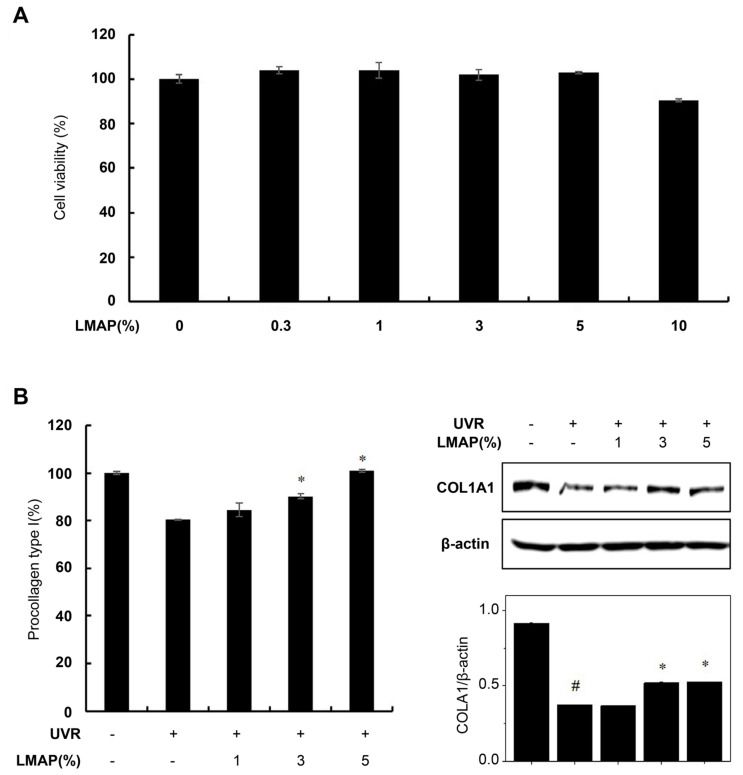
Effect of LMAPs on the expression of procollagen type I in fibroblasts. LMAP treatment had no effect on fibroblast viability (**A**). LMAPs increased the secreted protein and cellular protein levels of type I collagen in UVR-induced fibroblasts (**B**). Data are presented as mean ± S.D. *n* = 3; ^#^ *p* < 0.05 vs. untreated control, * *p* < 0.05. LMAP: low-molecular-weight anionic peptide; UVR: UVA 2 J/cm^2^ + UVB 15 mJ/cm^2^.

LMAP treatment had no effect on the viability of fibroblasts (Figure 6A). Type I collagen levels were significantly decreased in the UVR-irradiated fibroblasts compared with that in the negative control (*p* < 0.05; Figure 6B). However, LMAP treatment of the UVR-irradiated fibroblasts significantly increased the levels of type I collagen (*p* < 0.05; Figure 6B).

**Figure 7:**
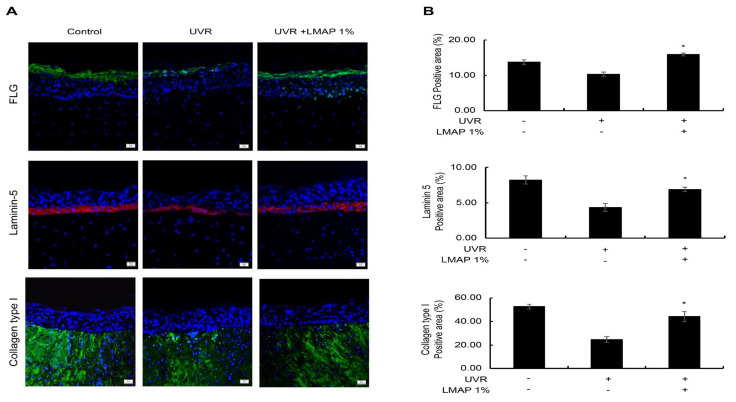
Effects of LMAPs on wrinkle formation in the reconstructed skin model following UVR treatment. (**A**) Immunofluorescence staining images and (**B**) Calculating graph. LMAPs inhibited wrinkle formation by increasing the expression of FLG, laminin-5, and type I collagen in a UVR-induced photoaging skin model. Data are presented as mean ± S.D. *n* = 3; * *p* < 0.05. LMAP: low-molecular-weight anionic peptide; UVR: UVA 2 J/cm^2^ + UVB 15 mJ/cm^2^. Scale bar, 50 μm.

The expression of filaggrin (FLG) in the epidermis was significantly decreased following UVR irradiation compared with that in the negative control (*p* < 0.05; Figure 7). This decrease in expression was rescued by LMAP treatment (*p* < 0.05; Figure 7), indicating its ability to restore moisturization by enhancing the barrier function and natural moisturizing factor (NMF) of the skin, which were reduced by UVR irradiation.

The expression level of laminin-5 in the dermal–epidermal junction (DEJ) was significantly reduced by UVR irradiation (*p* < 0.05; Figure 7) and was rescued by LMAP treatment (*p* < 0.05; Figure 7). These results indicate that LMAP treatment could prevent wrinkle formation by strengthening the stability and functionality of the basement membrane.

The expression of type I collagen, which was decreased in the dermal layer of the skin tissue by UVR irradiation (*p* < 0.05; Figure 7), was rescued by LMAP treatment (*p* < 0.05; Figure 7). These results indicate the efficiency of LMAPs in preventing wrinkle formation by increasing the synthesis of type I collagen in the dermal layer.

## 3. Discussion

The present study is the first to confirm the beneficial effects of epidermal LMAP treatment in mitigating both melanogenesis and wrinkle formation in a reconstructed skin model of UVR-induced photoaging. The LMAP treatment directly scavenged excessive intracellular ROS, thereby protecting keratinocytes from ROS-induced damage, and inhibited the secretion of inflammatory cytokines by keratinocytes.

We aimed to develop a safe and biologically active functional material using discarded byproducts of citron seeds. The washed and dried citron juice byproduct was hydrolyzed using a *Bacillus*-derived enzyme to obtain low-molecular-weight peptides (less than 500 Da). Furthermore, these peptides were separated into LMAPs with an external charge of −0.84 mV using a simple cation exchange resin method.

Keratinocytes form the outermost layer of human skin, which contains 95% of the cells in the epidermis. UVB exposure induces oxidative stress in keratinocytes [19], and increased production of ROS is deleterious to keratinocytes [19]. However, previously, peptides from seaweed [20], a frog peptide [16], and a porcine placenta peptide [17] were reported to prevent the photoaging of skin by inhibiting ROS accumulation. Also, a peptide from tilapia gelatin hydrolysate inhibited ROS production by enhancing the levels of antioxidant factors superoxide (SOD) and glutathione (GSH) in UVB-irradiated HaCaT cells [18]. Furthermore, in this study, cellular ROS levels were significantly enhanced by UVR irradiation, but LMAPs directly removed the excessive intracellular ROS and increased the viability of UVR-irradiated keratinocytes.

UVB-induced ROS production promotes the secretion of inflammatory cytokines, which eventually results in cellular senescence and the degeneration of ECMs in the skin [21,22]. Therefore, treatment with antioxidative and anti-inflammatory agents is the main strategy for preventing and treating UVR-induced skin photoaging. In this study, we found that LMAPs strongly decreased the over-production of inflammatory cytokines (IL-6 and TNF-α) in UVR-irradiated keratinocytes. Therefore, the protective effect of LMAPs in UVR-induced photoaging may be related to its anti-inflammatory properties via the direct removal of excessive intracellular ROS.

In a full-layer 3D reconstructed skin model, human-derived primary keratinocytes and fibroblasts formed a multilayered model of the human dermis and epidermis [23]. It consisted of basal, spinous, granular, and keratinized epidermal layers organized in a manner similar to that in human skin [23]. The dermal layer was composed of human dermal fibroblasts embedded in a collagen matrix [24]. A full-layer 3D reconstructed skin model with a well-developed membrane structure is a realistic model for studying the effects of UVR irradiation and LMAP treatment on the skin [24].

Human skin pigmentation is regulated by complex and intricate interactions between melanocytes and keratinocytes in the epidermis, with a number of factors secreted by keratinocytes being involved [25,26]. The POMC-derived hormones fibroblast growth factor 2 (FGF2), endothelin-1 (ET-1), and prostaglandin E2 (PGE_2_) are representative cytokines secreted by keratinocytes that stimulate melanocytes to enhance melanogenesis [26]. Additionally, IL-6 stimulation upregulates FGF2 secretion in keratinocytes, myeloma cells, and basal cells through the IL-6/STAT3 pathway [27]. Our results showed that LMAPs downregulate IL-6 secretion from UVR-irradiated keratinocytes. Therefore, it is possible that LMAPs reduce FGF2 secretion through the IL-6/STAT3 pathway. However, this hypothesis needs to be confirmed by further studies, as does the mechanism of their influence on other paracrine factors such as POMC, ET-1, and PGE_2_. Several types of enzymes are involved in the synthesis of melanin in the skin, and they are activated by α-MSH, ET-1, and PGE_2_ [28]. Among the enzymes involved in melanin synthesis, TRP-2 converts DOPA-chrome into 5,6-dihydroxyindole 2-carboxylic acid (DHICA). Another enzyme, TRP-1, oxidizes DHICA into indole-5-6-quinone carboxylic acid; however, its function in humans is poorly understood [28]. Therefore, the enzyme involved in the synthesis of brownish DHICA-rich melanin in the human skin is TRP-2. In this study, a Fontana–Masson-based melanin staining analysis of UV radiation-induced photoaging models revealed a significant LMAP-dependent decrease in melanin production and TRP-2 expression. These results suggest that LMAP treatment after the UVR irradiation of reconstructed skin reduces pigmentation by inhibiting TRP-2. Also, the melanin inhibition assay using α-MSH-induced B16F10 cells revealed that LMAPs significantly suppress melanin synthesis via the downregulation of melanogenesis regulators, including MITF, TYR, and TRP-2.

Major changes were observed in the dermis of photodamaged skin. Type I collagen is associated with skin aging and accounts for 90% of dermal collagen. It is an important protein, primarily produced by fibroblasts, that imparts elasticity and flexibility to the skin [29,30]. The treatment of fibroblasts with inflammatory cytokines, including TNF-α and IL-6, downregulated collagen synthesis by upregulating the expression of MMPs, leading to ECM deposition [30]. In this study, we treated the epidermis with LMAPs following UVR irradiation and observed a collagen type I expression increase in the dermis. This result suggests that changes in the dermis may have occurred through the inhibition of paracrine factors in the keratinocytes rather than through the LMAPs directly increasing collagen synthesis. The LMAPs inhibited the secretion of IL-6 and TNF-α from the UVR-irradiated keratinocytes. Therefore, it is possible that LMAPs increase collagen type I in the dermis by inhibiting IL-6 and TNF-α secretion from keratinocytes in the epidermis. Additionally, epidermal–dermal crosstalk has been implicated as the central mediator in various altered cutaneous processes [31]. UVB-irradiated epidermal keratinocytes influence fibroblasts in the dermal layer via signaling molecules. These signaling molecules trigger proteolytic enzymes responsible for changes in skin cells, thereby inducing an inflammatory response that alters the histological and molecular characteristics of skin, leading to photoaging [31]. Our results suggest that LMAP treatment of the epidermis mitigates the dermal–epidermal crosstalk following UV radiation.

FLG is essential for the production of NMF substances that maintain skin hydration and barrier function [32]. The stratum corneum is an important layer for maintaining moisture. However, as the skin ages, indicators of epidermal differentiation, including FLG, decrease, thereby reducing the moisture-holding ability of the stratum corneum [33]. The concentration of NMF components, which affect skin moisturization in the stratum corneum, is controlled by the amount of FLG; therefore, FLG is used as a biomarker for skin moisturization [34]. In this study, LMAP treatment increased FLG expression, which was attenuated by UV irradiation. An increase in FLG levels enhances the moisture content of the skin and strengthens the barrier function to prevent skin aging and wrinkle formation; therefore, the use of LMAP on UV-exposed skin is expected to strengthen the skin barrier function and to increase the moisture content.

The DEJ is composed of a complex network of proteins and proteoglycans that connects the epidermal and dermal layers and ensures important regulatory functions, such as determining the polarity of basal keratinocytes and promoting wound healing and re-epithelialization [35]. The major components of DEJ are laminins, type IV and VII collagen, perlecan, and nidogen [35]. Protecting the basement membrane from UV damage is considered one of the best methods for controlling skin aging [36]. In this study, LMAP treatment increased laminin-5 expression in the basement membrane, which was attenuated by UV radiation, indicating its potential to prevent wrinkle formation by strengthening the stability and functionality of the basement membrane.

Our study indicated that LMAP treatment protects against photoaging by suppressing melanogenesis and increasing the levels of FLG, laminin-5, and collagen type I. It acts by regulating the release of paracrine factors and the scavenging of ROS from keratinocytes. These results suggest that LMAPs are potential skin anti-photoaging agents for pharmaceutical and cosmetic applications.

This study has great significance in terms of ESG as it proposes a method to increase the added value of food-processing byproducts by increasing their usability through reprocessing. Bioactive peptides derived from the byproducts of food processing have a wide range of applications as resources in the food, pharmaceutical, and cosmetic industries. Therefore, further studies will be conducted to confirm the possibility of isolating bioactive peptides from other types of food-processing byproducts and the skin absorption of such peptides.

## 4. Materials and Methods

### 4.1. Materials

*Citrus junos* seed residue, a byproduct of the post-yuzu-juice-preparation process, was obtained from the Lotte R&D Center (Seoul, Republic of Korea). The following monoclonal antibodies were used: anti-tyrosinase-related protein-2 (TRP-2) (Abcam, Cambridge, UK), anti-collagen type IV (Abcam), and anti-filaggrin (Santa Cruz Biotechnology, Dallas, TX, USA). Anti-collagen type I antibody and all cell culture solutions were purchased from Invitrogen (Carlsbad, CA, USA).

For Western blotting, antibodies that act against tyrosinase, TRP-1, TRP-2, and MITF were purchased from Santa Cruz Biotechnology (Dallas, TX, USA), whereas antibodies that act against α-MSH, melanin, and β-actin were purchased from Sigma-Aldrich (St. Louis, MO, USA). 

### 4.2. Extraction and Separation of Low-Molecular-Weight Anionic Peptides

LMAPs were extracted and separated from *C. junos* TANAKA seeds, a byproduct in various primary processes. The washed and dried byproducts were hydrolyzed at 55 °C for 24 h using an enzyme derived from *Bacillus* sp. After filtration and sterilization of the reactant, an equal amount of ethyl alcohol was added to induce precipitation, followed by centrifugation; the supernatant containing low-molecular-weight peptides was concentrated. The concentrate was passed through an open column packed with a cation exchange resin to separate the LMAPs, which were then concentrated and used for further analysis.

### 4.3. Mass Spectrometry

Mass spectrometric measurements were performed at 25 °C using Microflex LRF MALDI-TOF mass spectrometry and Bruker UltrafleXtreme (Bruker Daltonics, Bremen, Germany). The intensity of each ion was calculated by adding the peak areas of the first isotope to that of the third isotope. Low-molecular-weight peptide and LMAP samples were dissolved in a 50% (*v*/*v*) methanol/water mixture. Thereafter, 1 μL of this sample solution was mixed with 1 μL of a sinapinic acid matrix (50% (*v*/*v*) in 0.1% TFA). The mixture was spotted onto a stainless-steel MALDI plate, dried, and analyzed.

### 4.4. Measurement of Zeta Potential

The zeta potential was measured at 25 °C using ELSZ-2000ZS (Otsuka Electronics, Osaka, Japan) equipped with a zeta potential cell. The low-molecular-weight peptide and LMAP samples were diluted to 1% (*w*/*w*) in distilled water and analyzed.

### 4.5. Cell Culture

Mouse melanoma B16F10 cells and human dermal fibroblasts (HDFns) were purchased from the American Type Culture Collection (ATCC; Manassas, VA, USA). B16F10 cells were cultured in Dulbecco’s modified eagle medium (Gibco, Paisley, UK) supplemented with 10% fetal bovine serum (Gibco), 100 U/mL penicillin, and 100 μg/mL streptomycin. The HDFn cells were cultured in F12: Dulbecco’s modified eagle medium (Gibco) supplemented with FBS and penicillin/streptomycin. The cells were subcultured every 2 to 3 days at 37 °C in a 5% CO_2_ incubator.

### 4.6. Evaluation of Cell Viability and Measurement of Cellular ROS

The cell viability of keratinocyte, B16F10, and HDFn treated with LMAP was detected using an MTT assay [21]. Briefly, the cells were seeded in 96-well plates, treated with LMAPs for 24 h, and incubated with MTT solution (100 µg/well) for 4 h. The formazan precipitate was dissolved in dimethyl sulfoxide, and MTT reduction was quantified by measuring the absorbance at 570 nm.

The intracellular ROS levels were measured as described previously [21]. The UVR-irradiated keratinocytes were incubated with LMAPs for 24 h and then in 10 μM H_2_DCFDA prepared in PBS at 37 °C for 30 min. Images were obtained using a Leica THUNDER Imager Tissue microscope analyzed using the ImageJ 1.47 Software (National Institutes of Health, Bethesda, MD, USA).

### 4.7. Enzyme Immunoassays for IL-6, TNF-α, and Procollagen Type I

The keratinocyte and HDFn culture media were collected and centrifuged at 13,000 rpm for 15 min to remove any debris. In the keratinocyte culture medium, the IL-6 and TNF-α levels were measured using ELISA kits, following the manufacturers’ instructions (Abcam, Cambridge, UK). In the HDFn culture medium, procollagen type I levels were assayed using the procollagen type I C-peptide ELISA kit (Takara, Otsu, Shiga, Japan). The levels were normalized by cell number.

### 4.8. Western Blotting

Protein extracts were prepared from B16F10 and HDFn cells by homogenization in a cell lysis buffer (Cell Signaling Technology, Danvers, MA, USA). The homogenates were centrifuged for 15 min at 2150× *g*, and the thus-obtained supernatants were used as total protein extracts. The protein concentration was measured using a bicinchoninic acid reagent (BioRad, Hercules, CA, USA). Equal amounts of protein were fractionated using SDS-polyacrylamide gel electrophoresis on a 10% gel at 100 V for 2 h. The resolved proteins were transferred onto polyvinylidene fluoride membranes (Millipore, Billerica, MA, USA) by electroblotting at 120 V for 1.5 h. The membranes were blocked with 5% skim milk for 1 h and then incubated with antibodies diluted in a 5% bovine serum albumin solution. After washing the membranes three times, the bands were detected using enhanced chemiluminescence (Amersham Pharmacia Biotech, Piscataway, NJ, USA) and quantified using the ImageJ 1.47 software.

### 4.9. Reconstructed Human Photoaging Skin Model

Normal human melanocytes (NHMs), normal human keratinocytes (NHKs), and fibroblasts were purchased from ATCC and cultured in a biosafety-level-2 system according to the standard protocol provided by ATCC. Dermal equivalents were obtained after contraction at 37 °C during 5 d of incubation with a mixture containing bovine type I collagen and fibroblasts, as previously described [37,38]. NHKs and NHMs were co-seeded at concentrations of 2 × 10^5^ and 2 × 10^4^ cells/well, respectively, on the top of the shrunken dermal equivalent, as previously described [39]. The culture was then immersed in a co-culture growth medium (CGM) comprising 80% KGM (keratinocyte growth medium) containing high calcium (1.5 mM) and 20% MGM (melanocyte growth medium) for three days to facilitate monolayer formation (immersion phase). In the post-immersion phase, cultures were raised to an air–liquid interface (ALI) and maintained for at least 12 d for stratification and differentiation of the keratinocytes in CGM supplemented with 50 mg/mL L-ascorbic acid and 10 ng/mL EGF.

RST was cultured in 6-well plates with CGM and irradiated with UVR (UVA 2 J/cm^2^ and UVB 15 mJ/cm^2^), and the RST epidermis was post-treated with citron seed anionic peptide via topical application for 5 d. On day 5 post-treatment, RSTs were collected and fixed in a 10% neutral formalin solution for 24 h, and then embedded in an OCT compound, and the frozen OCT blocks were cut into 12 μm sections.

### 4.10. Histological Analysis, Melanin Content, and Immunofluorescence Analysis

For the morphological evaluation, 12 μm RST sections were stained with hematoxylin and eosin (H&E) according to the standard protocol.

To quantify the melanin content, 12 μm thick RST sections were subjected to Fontana–Masson staining. The sections were then treated with a 2.5% aqueous silver nitrate solution for 10 min, 0.2% aqueous gold chloride for 1 min, and 5% aqueous sodium thiosulfate for 5 min. The melanin levels were normalized by counterstaining the epidermis with fast red.

To detect the expression of photoaging-related proteins, 12 μm frozen sections were hydrated in distilled water, subjected to antigen retrieval by heating in a citrate buffer (pH 6), and blocked with 3% bovine serum albumin (Sigma-Aldrich) prepared in PBS. The sections were incubated overnight in a humidified chamber at 4 °C with the following primary antibodies: anti-TRP-2 (1:100), anti-filaggrin (1:100), anti-collagen type I (1:500), and anti-laminin-5 (1:200). The sections were then incubated for 2 h with secondary antibodies, namely 1:1000 Alexa Fluor 488 goat anti-rabbit IgG (Abcam) or Alexa Fluor 555 goat anti-mouse IgG (Abcam); diluted in PBS; and mounted using a mounting medium containing 4′,6-diamidino-2-phenylindole (DAPI). Images were obtained using a Leica THUNDER Imager Tissue microscope and analyzed using the ImageJ Software.

### 4.11. Statistical Analysis

Immunostaining was semi-quantitatively analyzed based on areas in the photographs that showed staining using the ImageJ software. Antibody-positive areas were measured as follows: (1) three different sections from each RST (*n* = 3 tissue per group) were used, and (2) the percentage of stained area [(positive area/total area) × 100 (%)] was calculated. The total area included all skin layers. The results are presented as mean ± standard deviation (SD). Statistical analyses were performed using Microsoft Excel 2016 software.

## 5. Conclusions

Our findings suggest that LMAPs isolated from *C. junos* seed byproducts using a *Bacillus*-derived enzyme exert anti-photoaging effects in a UVR-treated reconstructed human skin aging model. The anti-photoaging effects of LMAPs were attributed to their ability to increase the levels of laminin-5, type I collagen, and filaggrin and to reduce TRP-2 and melanin content. LMAPs act by regulating the release of paracrine factors and the scavenging of ROS from keratinocytes. Thus, citron seed anionic peptides could be an effective ingredient for preserving skin health by preventing UVR-induced photoaging.

## Figures and Tables

**Table 1:** Zeta potential of peptides separated from *Citrus junos* TANAKA seeds.

Name	No.	Zeta Potential (mV)	Mobility (cm^2^/Vs)	E Field (V/cm)
Low-molecular-weight peptides	1	−1.08	−8.438 × 10^−6^	−16.19
	2	−0.63	−4.902 × 10^−6^	−16.19
	3	−0.81	−6.326 × 10^−6^	−16.19
	Average	−0.84	−6.555 × 10^−6^	−16.19
	STD	0.23	1.779 × 10^−6^	0.00
Low-molecular-weight anionic peptides	1	−8.96	−6.989 × 10^−5^	−16.03
	2	−8.77	−6.840 × 10^−5^	−16.02
	3	−8.43	−6.574 × 10^−5^	−16.02
	Average	−8.72	−6.801 × 10^−5^	−16.02
	STD	0.27	2.102 × 10^−6^	0.00

## Data Availability

The data can be made available by contacting the corresponding author.
